# Estradiol Downregulates MicroRNA-193a to Mediate Its Angiogenic Actions

**DOI:** 10.3390/cells14151134

**Published:** 2025-07-23

**Authors:** Lisa Rigassi, Mirel Adrian Popa, Ruth Stiller, Brigitte Leeners, Marinella Rosselli, Raghvendra Krishna Dubey

**Affiliations:** 1 Department of Reproductive Endocrinology, University Hospital Zurich, 8952 Schlieren, Switzerlandruth.stiller@usz.ch (R.S.); brigitte.leeners@usz.ch (B.L.); marinella.rosselli@usz.ch (M.R.); 2 Institute of Cellular Biology and Pathology Nicolae Simionescu, Romanian Academy, 050568 Bucharest, Romania; mirel.popa@icbp.ro; 3 Department of Pharmacology and Chemical Biology, University of Pittsburgh, Pittsburgh, PA 15219, USA

**Keywords:** estradiol, miRNA-139a, endothelial cells, angiogenesis, cardiovascular disorders

## Abstract

Estrogens regulate many physiological processes in the human body, including the cardiovascular system. Importantly, Estradiol (E2) exerts its vascular protective actions, in part, by promoting endothelial repair via induction of endothelial cell (EC) proliferation, migration and angiogenesis. Recent evidence that microRNAs (miRNAs) play an important role in vascular health and disease as well as in regulating Estrogen actions in many cell types. We hypothesize that E2 may mediate its vascular protective actions via the regulation of miRNAs. Following initial screening, we found that E2 downregulates the levels of miR-193a-3p in ECs. Moreover, miR-193a-3p downregulation by miR-193a-3p-antimir mimicked the effects as E2 on EC growth, migration, and capillary formation. Restoring miR-193a-3p levels with mimics after E2 treatment abrogated the vasculogenic actions of E2, suggesting a key role of miR-193a-3p in E2-mediated EC-growth-promoting effects. We further investigated the cellular mechanisms involved and found that miR-193a-3p inhibits angiogenesis by blocking phosphoinositide-3-kinase (PI3K)/Akt-vascular endothelial growth factor (VEGF) and Activin receptor-like kinase 1 (ALK1)/SMAD1/5/8 signaling in ECs, both pathways that are important in E2-mediated vascular protection. Additionally, using reverse transcription polymerase chain reaction (RT-PCR), we demonstrate that E2 downregulates miR-193a-3p in ECs via Estrogen Receptor (ER)α, but not ERβ or G protein-coupled estrogen receptor (GPER). Moreover, these actions occur post-transcriptionally, as the expression of pri-miR-193a-3p was not affected. The anti-angiogenic actions of miR-193a-3p were also observed in in vivo Matrigel implant-based capillary formation studies in ovariectomized mice where E2 induced capillary formation, and these effects were abrogated in the presence of miR-193a-3p, but not in the control mimic. Assessment of miR-193a-3p levels in plasma collected from in vitro fertilization (IVF) subjects with low and high E2 levels showed significantly lower miR-193a-3p levels in responders during the high E2 period. Hence, our findings provide the first evidence that miR-193a-3p mimic inhibits angiogenesis whereas its antimir is angiogenic. Importantly, E2 mediates its regenerative actions on ECs/capillary formation by downregulating endogenous miR-193a-3p expression. Both miR-193a-3p mimic or antimir may represent important therapeutic molecules to prevent or to induce endothelial function in treating pathophysiologies associated with capillary growth.

## 1. Introduction

Estrogens play a crucial role in the regulation of various physiological processes in the body. Among their many functions, they have been shown to exert significant protective effects against cardiovascular disease (CVD), one of the major causes of death and disability in Western civilization [1]. Epidemiological studies indicate a generally lower incidence of CVD in premenopausal women compared to in men of the same age. Furthermore, after menopause begins, the prevalence of CVD in women rises, as indicated by increased intimal thickening and plaque formation compared to premenopausal women [2], suggesting a possible role of ovarian hormones like estrogen. The association between vascular pathology and estrogen deficiency is further supported by the observation that postmenopausal women undergoing hormone replacement therapy have a lower risk of coronary disease [3,4] and overall mortality [5].

In vivo studies provide strong evidence for the beneficial effects of estrogens like estradiol (E2), which has been shown to prevent pathological processes associated with the development of hypertension, thrombosis, restenosis, cardiomyopathy, atherosclerosis, and other CVDs [6]. E2 influences cardiovascular health through multiple mechanisms, including the modulation of lipid profiles [7], enhancement of endothelial function [8], and reduction of inflammatory responses [9]. Moreover, E2 significantly influences endothelial cell (EC) growth and migration, which are critical processes for vascular repair and recovery, reendothelization, and angiogenesis [10,11,12].

E2 promotes endothelial cell proliferation by activating various signaling pathways, including the phosphatidylinositol 3-kinase (PI3K)/Akt pathway. This activation leads to increased cell survival and growth, contributing to the maintenance and repair of the endothelial lining. Furthermore, E2 enhances endothelial cell migration, a vital step in the formation of new blood vessels and the healing of damaged tissues. By stimulating both growth and migration of endothelial cells, E2 supports vascular health and helps in the recovery from vascular injuries/dysfunction, thereby playing a protective role in cardiovascular function [13].

Since estrogens also play an essential role in the development and malignant progression of multiple cancers [14], understanding the cardioprotective properties of E2 and the underlying molecular mechanisms is essential for developing targeted therapies and improving cardiovascular outcomes, particularly in postmenopausal women who experience a natural decline in estrogen levels.

MicroRNAs (miRNAs) are small, endogenous non-coding RNAs that play a crucial role in the regulation of gene expression by targeting messenger RNAs (mRNAs) for degradation or translational repression, thus modulating many biological processes including proliferation and migration, which play important roles in the pathophysiology of several diseases, including cardiovascular disorders [15,16,17]. Indeed, many miRNAs are dysregulated in diseased vessels, hence providing evidence of a key role in vascular remodeling associated with CVD [18,19,20].

MiRNAs represent a new regulatory mechanism of estrogen vascular action. Indeed, E2 has been shown to modulate the expression of various miRNAs in different human cell lines and tissues [21], thereby influencing numerous cellular processes, including those related to cardiovascular health [22]. Moreover, recent findings provide evidence that miRNAs may play an important role in meditating the protective effects of E2 in vascular cells [23]. For instance, E2 has been shown to upregulate miRNAs that promote endothelial cell proliferation and migration, such as miR-21 and miR-126, which enhance angiogenesis and vascular repair by targeting specific genes involved in cell cycle regulation and migration pathways [24,25]. In a previous screening of hormone-regulated miRNAs in endothelial progenitor cells (EPCs), miR-193a-3p was differentially regulated by E2 (unpublished data) and androgens. Moreover, recent findings demonstrate that miR-193a-3p influences endothelial cell growth and capillary formation and abrogates E2-induced proliferation of vascular ECs [26].

In the present study, we hypothesize that estrogen may, in part, mediate its growth and angiogenesis, promoting actions by downregulating miR-193a-3p formation in vascular ECs. To test our hypothesis, using cell proliferation, scratch assays, and capillary formation assays we investigated: the impact of miR-193a-3p mimic and antimirs on EC growth and function; the impact of E2 on miR-193a-3p production in ECs; the specific role of estrogen receptors (ER) α, β, and G protein-coupled estrogen receptor (GPER) in mediating the effects of E2 on miR-193a-3p synthesis; whether E2 induces angiogenic actions by blocking miR-193a-3p actions or by inhibiting its production; whether miR-193a-3p mediates its actions on ECs via key intracellular mechanisms (Activin receptor-like kinase 1 (ALK1)–SMAD1/5/8–inhibitor of differentiation 1 (ID1) pathway and the phosphoinositide-3-kinase (PI3K)/Akt–vascular endothelial growth factor (VEGF) pathway) that regulate angiogenesis; whether the in vitro actions of miR-193a-3p-mimic are also reproduced in vivo; whether changes in E2 levels influence miR-193a-3p levels in humans; and whether 2-methoxyestradiol, a growth inhibitory metabolite of E2, differentially modulates miR-193a-3p in ECs.

## 2. Materials and Methods

### 2.1. Cell Culture

Human Umbilical Vein ECs (HUVECs, Lonza, Walkersville, MD, USA; CC-2517) were cultured up to passage 10 with DMEM/F12 (Sigma-Aldrich, St. Louis, MO, USA; D-6434) supplemented with AA, LSGS (Life Technologies, Carlsbad, CA, USA; S-003-10), and 10% FCS (GE Healthcare, Hatfield, UK; SH30070). Cells were cultured in 75 cm^2^ flasks (TPP, Trasadingen, Switzerland) under standard tissue culture conditions (37 °C, 5% CO_2_). Upon confluence, cells were washed twice with HBSS -Ca^2+^/-Mg^2+^ (Bioconcept, Allschwil, Switzerland; 3-02K34-I) and incubated for 3 min at 37 °C with trypsin (Sigma-Aldrich, St. Louis, MO, USA; T-3924) diluted 1:4 in HBSS -Ca^2+^/-Mg^2+^. An equal amount of growing media was added to neutralize the reaction prior to centrifugation at 1200 rpm for 5 min. Cells were resuspended in fresh media and either plated in new 75 cm^2^ flasks at a split ratio of 1:4 or seeded onto tissue culture dishes or multiwall plates (BD, Franklin Lakes, NJ, USA).

### 2.2. Transfection of HUVECs with MicroRNA-193a Mimic and Antimir

HUVECs were plated in growth medium and allowed to recover for at least 24 h. Cells were transfected with has-miRNA-193a-3p (mature sequence 5′-AAC UGG CCU ACA AAG UCC CAG U-3′) AccuTarget microRNA Mimic or Antimir (Bioneer, Oakland, CA, USA; SMM-001 and SMI-001) and their respective negative controls (AccuTarget Negative controls Mimic or Antimir; Bioneer, Oakland, CA, USA; SMC-2001 and SMC-2101). Transfection was performed using Lipofectamine2000 (Life Technologies, Carlsbad, CA, USA; 11668019). Briefly, an appropriate amount of Lipofectamine2000 was diluted in serum and antibiotic-free DMEM/F12 in one tube (solution A), while microRNA oligonucleotides were diluted with the same media in a different tube (solution B). The solutions were incubated for 5 min at room temperature before being equally mixed (A + B) and incubated for 20 min at room temperature to allow the microRNA olignonucleotides−Lipofectamine2000 complexes to form. The cells were rinsed with serum- and antibiotic-free DMEM/F12, and the microRNA oligonucleotides−Lipofectamine2000 complexes were added to each well at a final mimic or antimir concentration of 25 nM. Mock transfection was carried out as described above but with the omission of the microRNA oligonucleotides. The transfection media was removed 5–6 h post-transfection and replaced by either the respective regular culture media or treatments.

### 2.3. Transfection Efficiency

Transfection efficiency was determined using miRIDIAN control mimic and antimir labeled with Dy547 (Dharmacon, Lafayette, CO, USA; CP-004500-01-05 and IP-004500-01-05). The transfected cells were stained with 0.5 ug/mL Hoechst33342 (Life Technologies, Carlsbad, CA, USA; H3570) and incubated for 30 min prior to detection under a fluorescence microscope (Olympus, Wallisellen, Switzerland). Quantitation of transfection efficiency was achieved employing a GUAVA easyCyte HT Flow Cytometer (GUAVA, Hayward, CA, USA). Transfected cells were detached by trypsinization, centrifuged and resupsended in 300 µL sample buffer (1 g/L glucose in PBS) before acquisition of the fluorescent signal. Cells transfected with an unlabeled mimic and antimir were used as negative controls.

### 2.4. Expression of Pri- and Mature MiRNA by qRT-PCR

HUVECs were treated 24 h with 10 nM 17-β-Estradiol (E2, Steraloids, Newport, RI, USA; E950) in DMEM/F12 supplemented with AA and 1% BSA (Sigma-Aldrich, St. Louis, MO, USA; T-9647), prior to RNA extraction and RT-qPCR. DMSO (Sigma-Aldrich, St. Louis, MO, USA; D2650) at a final concentration of 0.1% was applied as a vehicle-treated control.

The role of ERs was investigated using ER agonists PPT (Tocris, Bristol, UK; 1426), DPN (Tocris, Bristol, UK; 1494), and G1 (Calbiochem, Darmstadt, Germany; 371705) at 100 nM each. ERα antagonists MPP (500 nM, Tocris, Bristol, UK; 1991) and ICI 182-780 (ICI, 1 μM, Tocris, Bristol, UK; 1047) were added 1 h prior to E2 or PPT. To study the role of estrogen metabolites, the cells were treated with 0, 0.1, 1, and 3 µM 2-Methoxyestradiol (2ME, Steraloids, Newport, RI, USA) for 24 h, prior to RNA extraction and miR-193a-3p expression determination by qRT-PCR.

MicroRNA-193a overexpression and downregulation were assessed 24 h, 48 h, and 72 h after transfection with the mimic, the antimir, and their respective negative controls. For pri-miRNA expression analysis, the cells were stimulated for 6 and 24 h with or without 10 nM E2 in starving media.

Total RNA, including small RNAs, was extracted using the Quick-RNA MiniPrep Kit (ZymoResearch, Irvine, CA, USA; R1055), according to the manufacturer’s protocol, and quantified by the absorbance at 260 nm using the Infinite200 NanoQuant (Tecan, A). RT-PCR for miRNA-193a was performed using a TaqMan microRNA assay (Life Technologies, Carlsbad, CA, USA; 4440887, Assay ID 000524, mature sequence 5′-AAC UGG CCU ACA AAG UCC CAG U-3′) which provided microRNA-specific RT primers as well as primers and probes for amplification and detection of the microRNA. Briefly, single-stranded cDNA was synthetized from 10 ng total RNA in 15 μL reaction volume with the TaqMan microRNA Reverse Transcription Kit (Life Technologies, Carlsbad, CA, USA; 4366597). Each 15 μL reaction contained 1 mM dNTP mix, 50 U MultiScribe Reverse Transcriptase, 1× Reverse Transcription Buffer, 0.3 U RNase Inhibitor, and 1× microRNA -specific RT primers. The reaction was incubated at 16 °C for 30 min, followed by 30 min at 42 °C, and then inactivated at 85 °C for 5 min. Samples were chilled on ice and diluted by addition of 75 µL DEPC-treated water (Life Technologies, Carlsbad, CA, USA; AM9916). Amplification and detection of the specific products were performed on a Bio-Rad CFX Real-Time PCR Detection System (Biorad, Reinach, Switzerland). PCR reaction included 5 μL 2× TaqMan Fast Advanced Master mix (Life Technologies, Carlsbad, CA, USA; 4444964), 0.5 μL each 20× TaqMan microRNA Assay mix, 0.5 µL DEPC treated water, and 4 μL cDNA. The PCR reaction plate was run as follows: 2 min at 50 °C and 20 s at 95 °C, followed by 40 cycles of 95 °C for 3 s and 60 °C for 30 s. As internal controls, U48 (Life Technologies, Carlsbad, CA, USA; 4440887, Assay ID 001006, mature sequence: 5′-GAT GAC CCC AGG TAA CTC TGA GTG TGT CGC TGA TGC CAT CAC CGC AGC GCT CTG ACC-3′) and U49 (Life Technologies, Carlsbad, CA, USA; 4440887, Assay ID 001005, mature sequence: 5′-CAC TAA TAG GAA GTG CCG TCA GAA CGA TAA CTG ACG AAG ACT ACT CCT GTC TGA TT-3′) were used for microRNA template normalization.

For pri-miRNA expression, the TaqMan High-Capacity cDNA Reverse Transcription Kit (Life Technologies, Carlsbad, CA, USA; 4427012, Assay ID Hs03303307_pri) was used to synthetize single-stranded cDNA from the extracted RNA. Briefly, a 20 µL reaction mixture contained 1× Reverse Transcription Buffer, 1× RT Random Primers, 4 mM deoxynucleotide (dNTP) Mix, 50 U MultiScribe Reverse Transcriptase, nuclease-free water, and 10 µL of 20 µg/µL RNA. The reaction was incubated at 25 °C for 10 min, followed by 120 min at 37 °C, and then inactivated at 85 °C for 5 min. cDNA was stored at −20 °C or placed on ice for immediate procession with qPCR. Amplification and detection of the specific products were performed on a Bio-Rad CFX Real-Time PCR Detection System. PCR reaction included 5 μL 2× TaqMan Fast Advanced Master mix, 0.5 μL each 20× TaqMan pri-miRNA Assay mix, 2.5 μL DEPC treated water, and 2 μL cDNA. The PCR reaction plate was run as follows: 2 min at 50 °C and 20 s at 95 °C, followed by 40 cycles of 95 °C for 3 s and 60 °C for 30 s. As an internal control, GAPDH (Life Technologies, Carlsbad, CA, USA; 4331182, Assay ID Hs99999905_m1) and hPRT1 (Life Technologies, Carlsbad, CA, USA; 4331182, Assay ID Hs99999909_m1) were used for pri-miRNA template normalization.

The relative gene expression was assessed by comparing cycle times (Ct) through the CFX Connect software version1.4.1 (Biorad, Reinach, Switzerland). Target Ct values were normalized by subtracting the internal control values, which provided the ΔCt value. The relative expression level between treatments was calculated using the following equation: 2^−(ΔCt sample−ΔCt control)^.

### 2.5. HUVEC Growth Studies: Microvessel Formation, Migration, and Proliferation

HUVECs were treated with E2 (10 nM), TGF-β1 (1 ng/mL; R&D Systems, Minneapolis, MN, USA; 240-B), PI3K inhibitor LY0294002 (LY, 5 μM; Merck, Darmstadt, Germany; 440202), ALK1 specific neutralizing antibody (ALK1fc, 100 ng/mL; R&D Systems, Minneapolis, MN, USA; 370-AL-100), VEGF (100 ng/mL; Sigma-Aldrich, St. Louis, MO, USA; V-7295), and VEGF-A specific neutralizing antibody (VEGFab, 500 ng/mL; R&D Systems, Minneapolis, MN, USA; MAB293) in starving media (DMEM/F12 supplemented with AA and 1% BSA).

For the microvessel formation, cells were collected by trypsinization and incubated at 37 °C for 30 min with or without 10 nM E2 in 1 mL DMEM/F12 supplemented with AA and 0.4% BSA. Aliquots of 50 μL, each containing 4000 cells, were seeded onto an angiogenesis u-slide coated with 10 μL Matrigel (BD Biosciences, Franklin Lakes, NJ, USA; 356237) and allowed to form microvessels overnight. HUVECs transfected with miRNA-193a mimic and antimir and their respective negative controls were kept for 24 h in growing media post-transfection before being collected. Microvessel formation was imaged using a bright field microscope (Olympus, Wallisellen, Switzerland). Five pictures were taken for each well at a 10× magnification. Microvessel length was determined using Xcellence Pro software v1.2 (Olympus, Wallisellen, Switzerland).

Cell migration was determined by a scratch wound assay. HUVECs at 100% confluence in 6-well plates were wounded with a sterile pipette to generate a cell-free gap. The cells were then washed twice with PBS and photographed before treatment at a 4× magnification using a bright field microscope (Olympus, Wallisellen, Switzerland) to record the wound width after the scratch (T0). For the investigation of miRNA-193a’s role, cells were grown to 80% confluence before transfection and allowed to grow to confluency 48 h post-transfection before scratching and treatment. Twenty-four hours later (T24), photographs were taken again. Wound closure was determined using Xcellence Pro software v1.2 (Olympus, Wallisellen, Switzerland): wound area at T0 was subtracted by wound area at T24 and divided by wound area at T0 to normalize for the differences in wound size.

HUVEC proliferation was determined by cell counting. The cells were plated in 12-well plates and growth-arrested in starving media for 24 h before treatment. For the investigation of miRNA-193a’s role, cells were starved overnight prior to cell transfection and treated 5–6 h post-transfection with or without PDGF-BB and E2. After three days, cells were dislodged by trypsinization and counted in a Coulter counter.

### 2.6. Protein Expression Studies

For protein expression and phosphorylation analysis, HUVECs were serum-starved for 24 h and stimulated with 10 nM E2 for 45 min. Mimic- and antimir-transfected cells were kept in growing media 48 h post-transfection prior to cell lysis.

To test transfection efficiency, HUVECs were lysed for 24 h, 48 h, and 72 h after transfection with the GAPDH-targeting AccuTarget microRNA Mimic Positive Control (Bioneer, Oakland, CA, USA; SMC-1001).

Cells were washed with cold PBS, lysed in 60 μL Lysis Buffer (Cell Signaling Technology, Danvers, MA, USA; 9803) and homogenized for 2–10 s by sonication. Protein concentration was determined using the BCA Protein Assay Kit (Pierce Biotechnologies, Rockford, IL, USA; 23227) according to the manufacturer’s protocol.

Equal amounts of proteins were diluted with Lämmli Sample Buffer (4×, Biorad, Reinach, Switzerland; 161-0747) and DTT (20×, Fermantas, Glen Burnie, MD, USA; R0891), incubated for 5 min at 95 °C and subjected to SDS-PAGE. Standard Western blot analysis was conducted using anti-Akt (Cell Signaling Technology, Danvers, MA, USA; 9727), anti-phospho-Akt (Ser473; Cell Signaling Technology, Danvers, MA, USA; 9271), anti-ALK1 (Santa Cruz Biotechnology, Santa Cruz, TX, USA; sc-28976), anti-GAPDH (Abcam, Cambridge, UK; ab9484), anti-ID1 (Santa Cruz Biotechnology, Santa Cruz, TX, USA; sc-488), and anti-VEGF-A (Santa Cruz Biotechnology, Santa Cruz, TX, USA; sc152) antibodies. An anti-β-actin antibody (Sigma-Aldrich, St. Louis, MO, USA; A5441) was used as a loading control.

### 2.7. Ovariectomized Model C57Bl6 and Matrigel Plug Assay

Mice were bred and maintained under SPF conditions within the Animal Department of the “Nicolae Simionescu” Institute of Cell Biology and Pathology and were obtained from The Jackson Laboratory (Bar Harbor, ME, USA). The animal’s diet was ad libitum and was observed daily for abnormal clinical signs.

All in vivo experiments involving the animal study were approved by the Ethics Commission of the Institute of Biology and Cell Pathology “Nicolae Simionescu” and performed in accordance with the Guide to the Care and Use of Laboratory Animals published by the US National Institutes of Health (NIH 85-23, 1996 review). The mice (females) were divided into five groups: two control groups (positive: VEGF; negative: Matrigel only) and three experimental groups (Matrigel with E2 (100 nM), miRNA control (25 nM), and miRNA 193a-3p (25 nM)). All mice were ovariectomized prior to the experiment and used at 8–9 weeks age, with at least 5 mice per group.

C57Bl/6 females were anesthetized by intraperitoneal injection of a mixture of ketamine (90 mg/kg) and xylazine (10 mg/kg; K/X) in a ratio of 1/10. Confirmation of anesthesia was confirmed by reflex test (the lower limb of the animals was gently pressed with the researcher’s fingers). After the total confirmation of the anesthesia, the females were shaved in the dorsal region and cleaned with Betadine (100 mg/mL) before surgery. During the surgical intervention, the animals were positioned on a device with continuous heating, and the body temperature was continuously monitored and maintained at 37 °C.

After removal of both ovaries, the animals were allowed to wake up from anesthesia (about 1/2 h and kept at a temperature of 35 °C), then moved to a clean cage and kept separate in the first days after surgery.

The Matrigel^®^ plug/formation test is well established to assess the progression of angiogenesis and can be observed in a few days. The Matrigel was kept at −20 °C and thawed at 4 °C prior to use to become liquid, since at 37 °C (mouse body temperature) it turns into a solid gel. A total of 500 μL injections solution was used for each mouse. The injectable solution was a mixture of 450 μL of Corning^®^ Matrigel^®^ growth factor reduced basement membrane matrix (Corning Inc., New York, NY, USA) and 50 μL of lipofectamine plus miRNA or control miRNA with a final concentration of 25 nM. This whole procedure was performed on females aged 11–12 weeks and under ketamine−xylazine anesthesia, which was administered by subcutaneous injection in the dorsal surfaces. On day 7, Matrigel plugs were surgically removed from anesthetized animals. The excised plugs were subjected to light microscopy and hemoglobin determination.

### 2.8. Analysis of Hemoglobin from Matrigel Plugs by the Drabkin Method

Determination of hemoglobin from the microvessels embedded in the Matrigel plugs is an indicator of the number of blood vessels formed. After the surgical removal of Matrigel plugs, the plugs were weighed and homogenized in a hypotonic lysis buffer containing the Brij solution for 5–10 min on ice. Subsequently, the homogenate was subjected to 5000 rpm in a microcentrifuge for 5 min at 4 °C, and the derived supernatant was collected for hemoglobin measurement following the manufacturer’s protocol.

The supernatant was mixed with 500 μL of Drabkin’s reagent and kept at room temperature for 15–30 min. One hundred microliters of this mixture was transferred to the wells of a 96-well plate. The absorbance was measured at a wavelength of 540 nm. The hemoglobin concentration was related to the standard curve obtained previously according to the indications given by the manufacturer. The calculated values were normalized by dividing the percentage of hemoglobin by the weight of the plug and are expressed in g/dl of hemoglobin per milligram of Matrigel or by the weight of a weighed Matrigel formation.

### 2.9. Human Samples

Human plasma samples from patients undergoing hormone treatment (*n* = 7) were collected during modulation of ovarian function where E2 levels were regulated to be low and then induced (high). The amount of miR-193a-3p in the samples was determined using qRT-PCR as described by Kroh et al. [27] and using a miRNeasy Serum/Plasma kit from Qiagen, which employed a spike-in control for normalization of miRNA purification. The relative miR-193a-3p expression was assessed by comparing cycle times (Ct) through the CFX Connect software version 1.4.1 (Biorad, Reinach, Switzerland). Target Ct values were normalized by subtracting the internal control values, which provided the ΔCt value. The relative expression level between treatments was calculated using the following equation: 2^−(ΔCt sample−ΔCt control)^.

### 2.10. Statistical Analysis

Unless stated differently, all data are presented as mean ± standard error. Experiments were repeated at least three times. For statistical evaluation, Student’s *t* tests and ANOVA were used, and statistical significance (*p* < 0.05) was calculated using Fisher’s Least Significant Difference test.

## 3. Results

### 3.1. E2 Downregulates miR-193a but Not miR-146, miR-409-5p, and miR-494 in HUVECs

**Figure 1:**
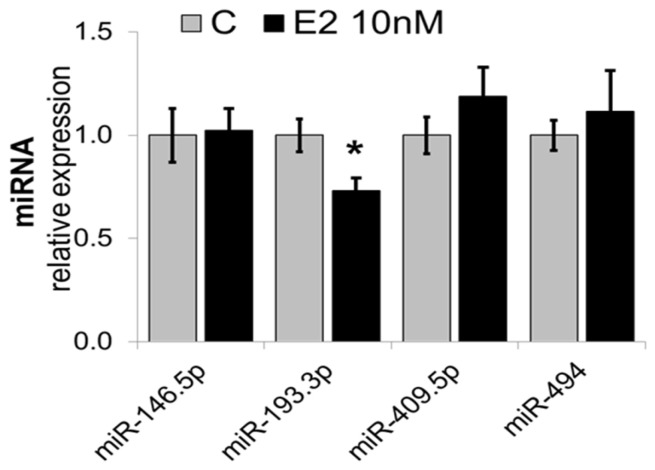
Differentially regulated miRNAs in HUVECs in response to Estradiol (E2; 10 nM). HUVECs were grown to 60% confluency in complete media prior to 24 h treatment with or without 10 nM E2. Total RNA was extracted, and relative microRNA expression levels were determined by RT-qPCR using TaqMan microRNA assays for miR-146a.5p, miR-193a-3p, miR-409.5p, and miR-494. The results were normalized to U48 and U49. * *p* < 0.05 vs. control.

Based on our previous findings that E2 modulates multiple miRNAs in EPCs, including miR-193a-3p (unpublished data), we assessed whether E2 similarly modulates the expression of highly regulated miRNAs in HUVECs (grown to 60% confluency), using RT-qPCR. Treatment of HUVECs with E2 selectively mimicked its modulatory effects on miR-193a-3p (Figure 1) in EPCs and downregulated the expression by 27% effects. The expression of miR-146-5p, miR-494, and miR-409-5p were not significantly modulated. Based on these findings, we selected miR-193a-3p and further assessed its role in mediating E2 induced angiogenic actions in HUVECs.

### 3.2. MiR-193a-3p Transfection Efficiency in HUVECS

**Figure 2:**
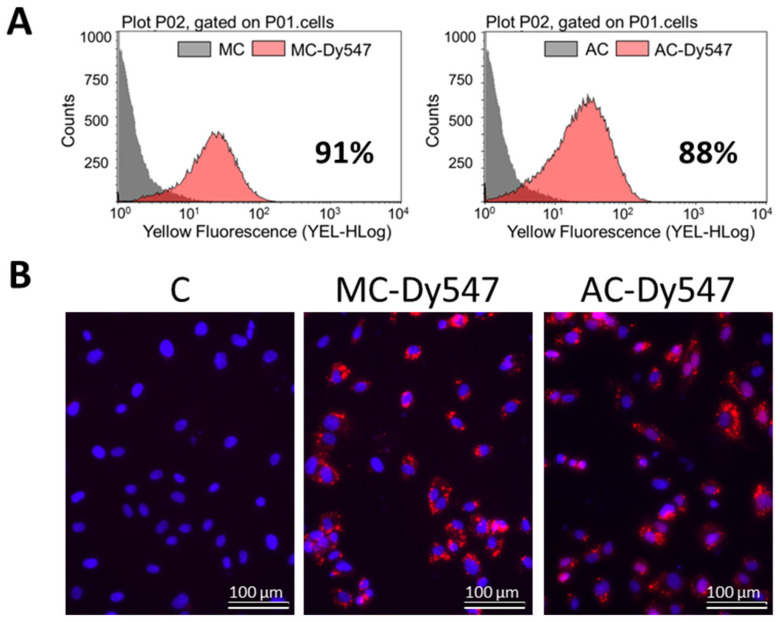
Transfection efficiency of miR-193a-3p in HUVECs. (**A**) Flow cytometric profile of the percentage of Dy547-positive cells with respect to the control cells, transfected with an unlabeled control mimic (MC) and a control antimir (AC). (**B**) Representative fluorescent photomicrographs of HUVECs transfected with Dy547-labeled MC and AC. Red indicates Dy547-labeled mimic or antimir; blue represents Hoechst33342 stain. Experiments were performed at least 3 times in triplicates.

To assess whether miR-193a-3p mediates the growth regulatory actions of E2 in HUVECs, we first established its transfection efficiency using a 25 nM Dy547-labeled control mimic (MC-Dy547) and antimir (AC-Dy547). After 6 h transfection, using Lipofectamine 2000 in the absence of antibiotics and serum, HUVECs were allowed to recover for 24 h in growth medium. Flow cytometry was used to quantify the percentage of Dy547-positive cells with respect to the control cells, which were transfected with an unlabeled control mimic (MC) and control antimir (AC) (depicted in Figure 2A), whereas fluorescence microscopy was used to take representative images (red indicates Dy547-labeled mimic or antimir; blue represents Hoechst33342 stain (Figure 2B)). Flowcytometric data revealed a 91% transfection efficiency with MC and an 88% with AC. Moreover, this outcome was reflected by the positive staining of transfected HUVECs.

**Figure 3:**
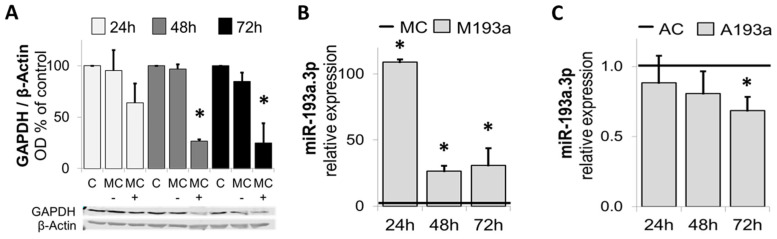
Confirmation of optimal transfection conditions. (**A**) Western blots of lysates from HUVECs transfected with 25 nM GAPDH-targeting positive control mimic (MC+), negative control (MC−), or mock transfected (C). Cells were kept in growing media for 24 h, 48 h, and 72 h prior to cell lysis and analysis of GAPDH protein expression using Western blot. β-Actin was used as a loading control. *n* = 3, * *p* < 0.05 compared to MC−. (**B**,**C**) Changes in miR-193a-3p expression in HUVECs by miR-193a-3p mimic and antimir, respectively. HUVECs were transfected with 25 nM miR-193a-3p mimic (M193a) or antimir (A193a) and the respective negative controls—MC (mimic) and AC (antimir). RNA was extracted after 24 h, 48 h, and 72 h prior to the determination of miR-193a-3p levels by RT-PCR. (*n* = 3). * *p* < 0.05 vs. negative control.

After assessing the transfection efficiency of miR-193a-3p in HUVECs, we further assessed optimal experimental conditions for transfection by means of a positive control mimic specifically targeting Glyceraldehyde-3-phosphate dehydrogenase (GADPH). Figure 3A shows HUVECs transfected with 25 nM GAPDH-targeting Positive Control Mimic (MC+), Negative Control (MC−), or mock transfected (C) were kept in growing media for 24 h, 48 h, and 72 h prior to cell lysis and analysis of GAPDH protein expression using Western blot. β-Actin was used as a loading control (*n* = 3, * *p* < 0.05 compared to MC−). Protein expression of GADPH was reduced by ≈ 40% (n.s.) 24 h post-transfection and by ≈ 75% (*p* < 0.05) at 48 h and 72 h after transfection with the positive control mimic.

Next, we investigated the alteration in miR-193a-3p by ectopic application of miR-193a-3p mimic and antimir. HUVECs were transfected with 25 nM miR-193a-3p mimic (M193a) or antimir (A193a) and the respective negative controls—MC (mimic) and AC (antimir). RNA was extracted after 24 h, 48 h, and 72 h prior to determination of miR-193a-3p levels by RT-PCR. As shown in Figure 3B,C, compared to the mimic control (MC), miR-193a-3p mimics significantly increased the levels of miR-193a-3p in HUVECs after 24 h, 48 h, and 72 h (Figure 3B). Moreover, transfection with miR-193a-3p antimir resulted in a time-dependent reduction in miR-193a-3p levels (Figure 3C), which was significant after 72 h (32 ± 9.78% reduction; *p* < 0.05). Values represent mean ± SEM (*n* = 3). * *p* < 0.05 versus negative control (MC or AC).

### 3.3. MiR-193a-3p Mimic Inhibits EC Microvessel Formation, Migration, and Proliferation Whereas Its Antimir Has Pro-Growth/Capillary-Stimulating Effects Similar to E2

**Figure 4:**
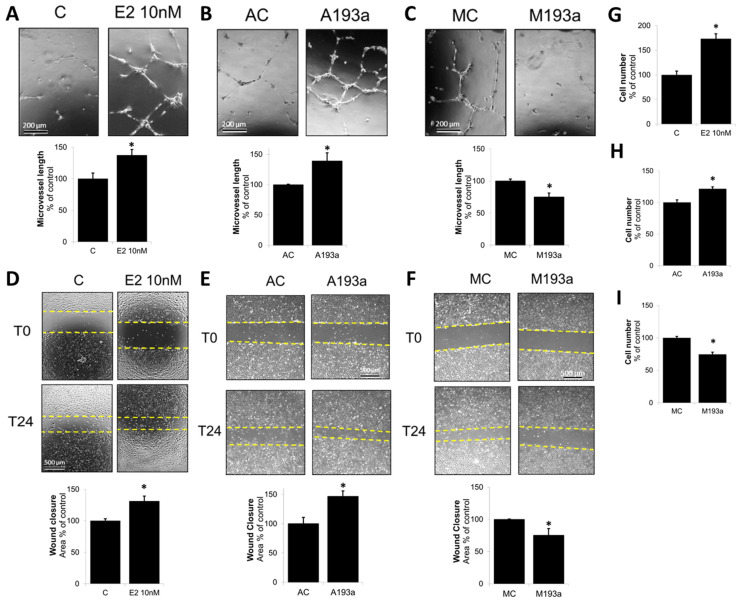
Downregulation of miR-193a-3p induces HUVEC microvessel formation, migration, and proliferation similar to E2, whereas miR-193a-3p upregulation has opposite effects. ECs were either treated with or without 10 nM E2 in starving media or transfected with miR-193a-3p antimir (A193a) or miR-193a-3p mimic (M193a) and their respective negative controls (AC and MC). (**A**–**C**) Microvessel formation was investigated using a Matrigel-based assay. Transfected cells were left in growing media for 48 h prior to seeding in serum-free media on Matrigel, while non-transfected cells were incubated for 30 min with or without 10 nM E2. HUVECs were allowed to form tube-like structures for 18 h before tube length was measured. Representative images for each condition are depicted. (**D**–**F**) Migration was investigated using a scratch wound assay. Transfected cells were allowed to grow for 48 h before the wound was made. Photomicrographs show representative images for each condition immediately after the scratch (T0) and 24 h later (T24). (**G**–**I**) Proliferation was assessed by counting the cells three days after transfection. The results are presented as mean ± SEM (*n* = 3). * *p* < 0.05 versus the respective control (C, AC, or MC).

Once the miR transfection conditions were optimized and established, we further assessed the modulatory impact of miR-193a-3p mimics (M193a) and antimirs (A193a) and their respective controls (MC and AC) on EC microvessel formation, EC migration (using wound closure assay), and cell proliferation. Panels A, B, and C of Figure 4 show representative photomicrographs and bar graphs for the effects of E2 (10 nM), A193a, and M193a on microvessel formation by ECs. Treatment of non-transfected ECs with E2 for 18 h induced capillary formation (Figure 4A). Moreover, the effects of E2 on microvessel formation were mimicked by ECs transfected with miR 193a antisense (A193a; Figure 4B), whereas inhibitory effects on capillary formation were observed in ECs transfected with miR-193a-3p mimic (M193a; Figure 4C). As shown in Figure 4A–C, compared to the respective controls, treatment with E2 or A193a induced microvessel formation by 137 ± 9% (*p* < 0.05 vs. C) and 139 ± 13% (*p* < 0.05 vs. AC), respectively, whereas M193a inhibited capiullary formation by 75 ± 6% (*p* < 0.05 vs. MC).

Next, we investigated the effects of E2, A193a, and M193a on EC migration using a scratch assay. As shown in Figure 4D–F, treatment with E2 induced EC migration to 131 ± 8% (*p* < 0.05 compared to untreated control (C)). Moreover, similar to our observations in capillary formation, transfection of ECs with AntimiR-193a-3p (A193a) significantly induced migration to 147 ± 9% whereas M193a significantly blocked EC migration to 75 ± 11% as compared to AC and MC, respectively (Figure 4E,F).

Since cell proliferation plays a key role in vascular repair and E2 has been shown to promote EC growth [12], we assessed how microRNA 193a antimirs (A193a) and mimics (M193a) influence EC cytokinesis. As shown in Figure 4G–I, treatment for 3 days with E2 increased EC cell number to 173 ± 10% (*p* < 0.05 vs. untreated control C; Figure 4G), and similar pro-proliferative effects were observed in ECs transfected with A193a (121 ± 3% *p* < 0.05 vs. AC; Figure 4H). Importantly, consistent with our observations in capillary formation and EC migration, transfection with mimic M193a inhibited cytokinesis to 74 ± 4% (*p* < 0.05 vs. MC; Figure 4I).

### 3.4. E2 Promotes EC Migration and Capillary Formation by Downregulating the Expression of miR-193a-3p

**Figure 5:**
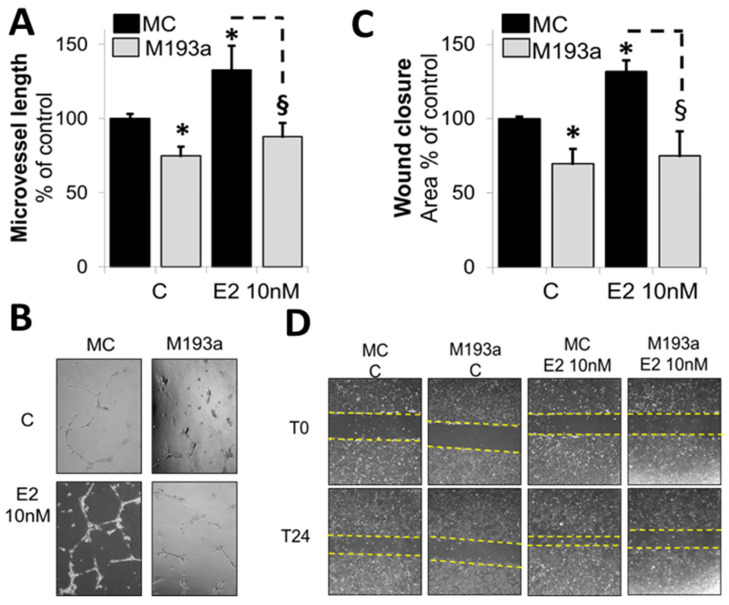
Ectopic expression of miR-193a-3p reverses the protective actions of E2 in HUVECs. HUVECs were transfected with the control and miR-193a-3p mimic (MC and M193a) and kept in growing media for 48 h prior to the determination of HUVEC function by Matrigel-based microvessel formation and scratch wound assays. (**A**,**B**) For microvessel formation, the cells were incubated for 30 min with and without 10 nM E2 in serum-free media before seeding on Matrigel and length measurement after 18 h. (**C**,**D**) For the migration, HUVECs were scratched before treatment with 10 nM E2 in serum-free media. Representative images of the scratch wound assay for each condition were taken immediately after the scratch (T0) and 24 h later (T24). The results are presented as mean ± SEM (*n* = 3). * *p* < 0.05 versus control (C or MC). § *p* < 0.05 between indicated treatments.

Our findings presented in Figure 1 and Figure 4 suggest that E2 potentially induces its stimulatory actions on EC capillary formation, EC migration, and proliferation by downregulating miR-193a-3p. To further prove this hypothesis, we assessed whether ectopic expression of miR-193a-3p mimic blocks the stimulatory actions of E2 on both capillary formation and EC migration. Treatment of normal ECs with E2 (10nM) induced capillary (Figure 5A,B) and promoted EC migration (Figure 5C,D). However, the stimuatory effects of E2 were completely abrogated in ECs trasfected with M193a (Figure 5A–D). These findings provide strong evidence in support of our hypothesis that E2 promotes EC growth by downregulating the expression of miR-193a-3p, an inhibitory miR in ECs.

### 3.5. MiR-193a-3p Does Not Affect EC Viability

**Figure 6:**
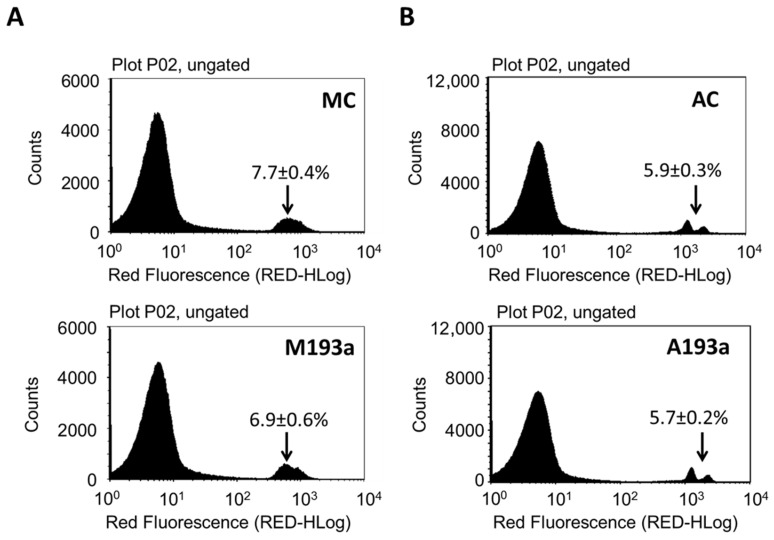
Alteration of miR-193a-3p levels does not affect ECs viability. HUVECs were transfected with the control mimic (MC), miR-193a-3p mimic (M193a, (**A**)), the control antimir (AC), and miR-193a-3p antimir (A193a, (**B**)) and cultured in growing media for 24 h after transfection. For PI staining, the cells were trypsinized, incubated for 5 min with 0.2 μg/mL PI and analyzed for viability with a flow cytometer. The percentage of dead cells is indicated. *n* = 3.

To make sure that the effects following transfection with miRNAs were not due to cell toxicity, the effects of both miR-193a-3p mimic (M193a) and antimir (A193a) were confirmed by assessing cell viability using flow cytometry. As shown in Figure 6, we observed no loss in cell viability in ECs transfected with M193a or A193a under our experimental conditions.

### 3.6. Intracellular Mechanisms of MiR-193a-3p-Mediated Vasculogenesis

**Figure 7:**
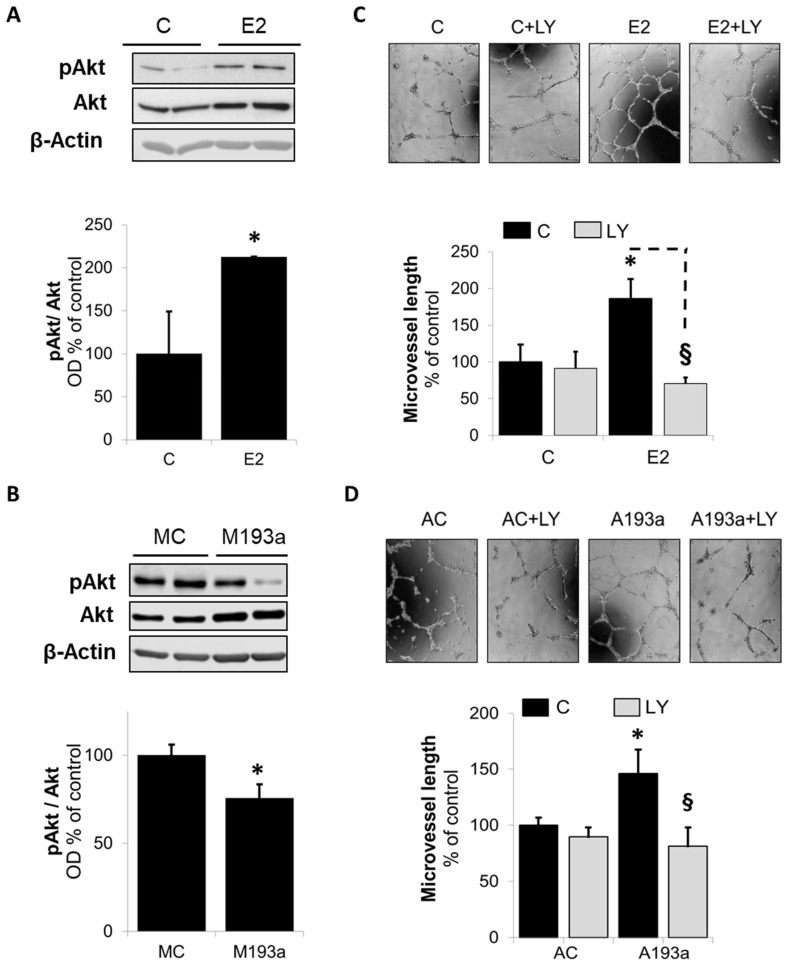
MiR-193a-3p modulates the E2-regulated PI3K/Akt pathway. (**A**,**B**) The representative blots and bar graphs of Akt phosphorylation (pAkt) after treatment with or without 10 nM E2 for 45 min or transfection with the control mimic (MC) and miR-193a-3p mimic (M193a). Total Akt and β-actin were used as loading controls. (**C**,**D**) The phosphoinositide-3-kinase (PI3K) inhibitor LY294002 (LY, 5 µM) abrogates the inducing effect of E2 and miR-193a-3p antimir on tube formation. Untransfected HUVECs and cells transfected with the control antimir (AC) and miR-193a-3p antimir (A193a) were pre-treated for 30 min with LY and subsequently for 30 min with or without 10 nM E2 and plated on the Matrigel. Tube length was measured after 16–18 h. Photomicrographs show representative images for each treatment. *n* = 3; * *p* < 0.05 compared to the respective control; § *p* < 0.05 as indicated.

To decipher whether miR-193a-3p is indeed involved in E2-mediated proangiogenic actions in ECs, we next investigated its impact on key pathways via which E2 triggers pro-angiogenic actions [26,28]. First, we looked at the modulation of Akt phosphorylation, a key signal transduction mechanism that feeds the downstream pro-angiogenic cascades involving VEGF. As shown in Figure 7A, challenging ECs with E2 for 45 min induced Akt phosphorylation (pAkt) to 212 ± 1% (*p* < 0.05 vs. control C; mean ± SEM; *n* = 3). To confirm the involvement of miR-193a-3p downregulation by E2 in mediating this action, we assessed Akt phosphorylation in ECs transfected with miR-193a-3p. As shown in Figure 7B, transfection with miR-193a-3p significantly inhibited the expression of pAkt to 75 ± 8% compared to the mimic control (*p* < 0.05 vs. MC). Moreover, inhibition of Akt phosphorylation with PI3K inhibitor LY294002 (5 µM) blocked capillary formation induced by both E2 and miR-193a-3p antisense (AC) in ECs (Figure 7C,D, respectively).

**Figure 8:**
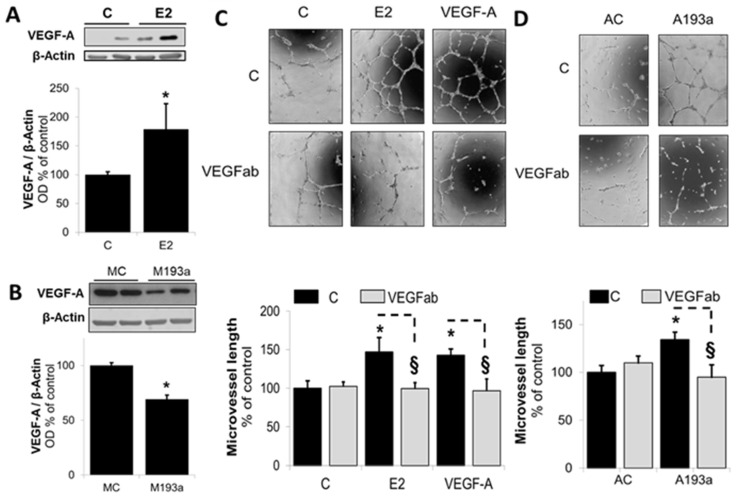
miR-193a-3p modulates E2-regulated VEGF signaling. (**A**,**B**) The representative blots and bar graphs of vascular endothelial growth factor (VEGF)-A protein expression after treatment with or without 10 nM E2 for 45 min or transfection with the control mimic (MC) and miR-193a-3p mimic (M193a). β-actin was used as a loading control. (**C**,**D**) The VEGF-A-specific neutralizing antibody (VEGFab, 500 ng/mL) abrogates the stimulatory effects of E2, VEGF-A, and miR-193a-3p antimir on tube formation. Non-transfected HUVECs and cells transfected with the control antimir (AC) and miR-193a-3p antimir (A193a) were pre-treated for 30 min with VEGFab and subsequently for 30 min with or without 10 nM E2 and VEGF-A (100 ng/mL) and plated on the Matrigel. Tube length was measured after 16–18 h. Photomicrographs show representative images for each treatment. *n* = 3; * *p* < 0.05 compared to the respective control; § *p* < 0.05 as indicated.

Downstream from activated Akt, VEGF plays a major role in mediating pro-angiogenic actions in ECs (ref). Importantly, E2 has been shown to mediate its pro-angiogenic actions via VEGF [28]. Hence, we further investigated whether downregulation of miR-193a-3p by E2 is involved in mediating E2-induced angiogenesis via VEGF. As shown in Figure 8A, treatment of ECs with E2 induced VEGF-A expression from 100 ± 5% in the control (C) to 179 ± 44% in E2-treated ECs (*p* < 0.05 vs. C). Importantly, as hypothesized, expression of VEGF-A was significantly decreased in ECs transfected with miR-193a-3p mimic (M193a). Compared to mimic control (MC), VEGF-A expression was decreased to 69 ± 4% in ECs transfected with M193a (Figure 8B). To confirm the functional relevance of alterations in VEGF-A expression and its role in mediating the actions of E2 on capillary formation via miR-193a-3p, we conducted capillary formation assays in the presence of VEGF-A-neutralizing antibodies (VEGFab). As shown in Figure 8C,D, E2- and miR-193a-3p antisense (A193a)-induced capillary formation was blocked in the presence of VEGF-A-neutralizing antibodies, suggesting that E2 mediates its capillary promoting actions in ECs, in part, via VEGF-A stimulation, and this involves downregulation of miR-193a-3p by E2.

**Figure 9:**
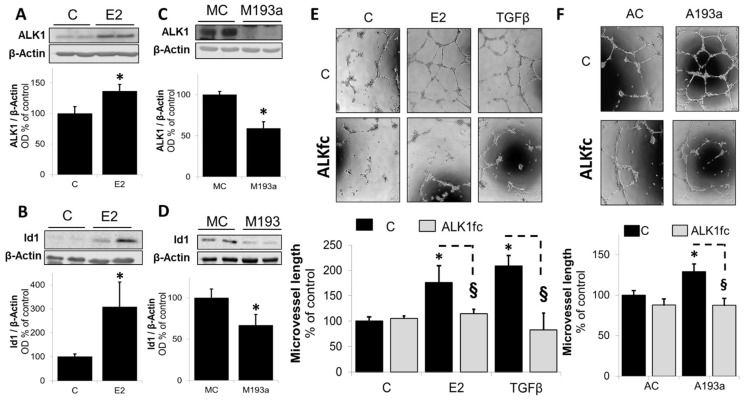
MiR-193a-3p modulates the E2-regulated ALK1/ID1 pathway. (**A**–**D**) Representative blots and bar graphs of Activin receptor-like kinase 1 (ALK1) and inhibitor of differentiation 1 (ID1) after treatment with or without 10 nM E2 for 45 min or transfection with the control mimic (MC) and miR-193a-3p mimic (M193a). Total Akt and β-actin were used as loading controls. (**E**,**F**) ALK1-specific antagonizing antibody (ALK1fc, 100 ng/mL) abrogates the effects of E2, transforming growth factor (TGF)-β and miR-193a-3p antimir on tube formation. Untransfected HUVECs and cells transfected with the control antimir (AC) and miR-193a-3p antimir (A193a) were pre-treated with ALK1fc for 30 min and subsequently with or without 10 nM E2 or TGF-β1 (1 ng/mL) for 30 min and plated on the Matrigel. Tube length was measured after 16–18 h. Photomicrographs show representative images for each treatment. *n* = 3; * *p* < 0.05 compared to the respective control; § *p* < 0.05 as indicated.

Apart from VEGF, other redundant mechanisms also regulate angiogenesis in parallel to each other. In this context, the transforming growth factor (TGF)-β pathway is well established to induce capillary formation/angiogenesis in ECs. It mediates its actions via ALK1 which targets Smad 1/5/8 to upregulate ID1, subsequently triggering angiogenesis. To assess whether E2 induces angiogenesis through activation of TGF-β and ID1 axis via miR-193a-3p downregulation, we investigated the impact of E2 and miR-193a-3p on both ALK1 and ID1 expression. Moreover, we further conformed their involvement by assessing whether E2- and miR-193a-3p antimir (A193a)-induced angiogenesis is blocked by an ALK1-specific antagonizing antibody (ALKfc). As shown in Figure 9, compared to the untreated control, treatment with E2 significantly increased ALK1 (panel A) and ID1 (panel B) expression in ECs to 137 ± 11% and 308 ± 103% (*p* < 0.05 vs. C), respectively. Moreover, in contrast to E2, ALK1 and ID1 expression was significantly decreased in ECs transfected with miR-193a-3p mimic (M193a; Figure 9C,D). Compared to ECs transfected with the mimic control (MC), M193a inhibited ALK1 and ID1 expression to 59 ± 8% and 67 ± 13% (*p* < 0.05 vs. MC), respectively. This observation is consistent with our results that E2 induces capillary formation by downregulating miR-193a-3p, which inhibits EC growth potentially via the ALK1/ID1 mechanism. This contention is further supported by our finding that E2- and miR-193a-3p antimir (AC)-induced capillary formation by ECs was blocked by ALKfc (Figure 9E,F). Taken together, our findings suggest that in ECs, miR-193a-3p mimic acts as an endogenous inhibitor angiogenesis and mediates its actions by inhibiting both the VEGF and ALK1/ID1 pathways. Importantly, E2 induces its pro-angiogenic actions by downregulating miR-193a-3p (mimic) expression and reversing its inhibitory actions on both the VEGF and ALK1/ID1 pathways.

### 3.7. E2 Downregulates MiR-193a-3p Expression via ER-α

**Figure 10:**
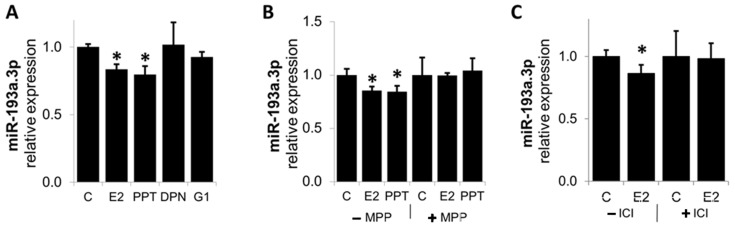
E2 modulates miR-193a-3p expression through ER-α in HUVECs. (**A**) HUVECs were treated with 10 nM E2 and ER agonists PPT (ER-α), DPN (ER-β), and G-1 (GPER) for 24 h, prior to RNA extraction and miR-193a-3p expression determination by qRT-PCR. (**B**,**C**) Cells were pre-incubated for 1 h with the ER-α specific antagonist MPP (500 nM) and the unspecific ER antagonist ICI 182-780 (1 µM). The results are presented as mean ± SEM (*n* = 3). * *p* < 0.05 versus control.

E2 has been shown to mediate its biological actions via nuclear and membrane ERα, Erβ, and GPER/GPR30 [29]. To assess the contribution of these receptors in mediating the effects of E2 on M193a downregulation in ECs, we assessed and compared the modulatory effects of E2 with those of ERα, Erβ, and GPER/GPR30 agonists—PPT, DPN, and G1, respectively. As shown in Figure 10A, treatment with E2 downregulated the expression of miR-193a-3p, and these effects were mimicked by ERα agonist PPT, but not by ERβ and GPER/GPR30 agonists—DPN and G1 respectively. To confirm the role of Erα, we assessed if E2 actions on miR-193a-3p were blocked by ERα antagonist—MPP. As shown in Panel B, MPP blocked the inhibitory actions of E2 as well as PPT on miR-193a-3p expression. Moreover, the effects of E2 were also blocked by ICI182780 (Panel C), a non-specific ER antagonist. These findings provide evidence that ERα plays a key role in mediating the angiogenic actions of E2 by facilitating the downregulation of miR-193a-3p.

### 3.8. E2 Does Not Influence Pri-MiR-193a-3p Expression

**Figure 11:**
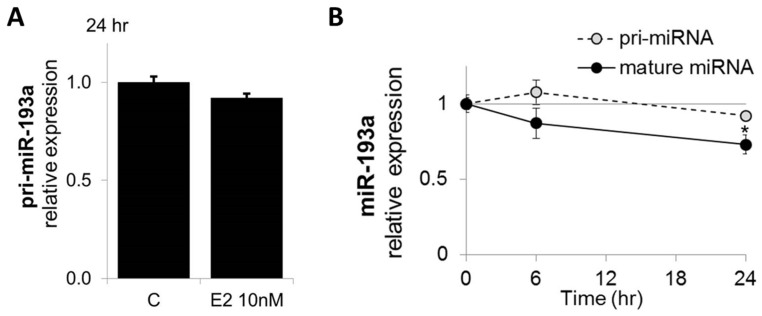
Expression of pri-miR-193a-3p and mature miRNA after E2 treatment. HUVECs were grown to 60% confluency in complete media prior to treatment with or without E2 (10 nM). (**A**) Pri-miR-193a-3p expression after 24 h treatment with E2. (**B**) Comparison of pri-miRNA and mature miRNA expression after 6 and 24 h treatment. Total RNA was extracted, and relative pri-miRNA expression levels were determined by RT-qPCR using TaqMan assays for pri-miR-193a-3p. The results from pri-miRNAs were normalized to GAPDH and Hypoxanthine-guanine phosphoribosyltransferase 1 (hPRT1) mRNAs. Mature miRNA expression was normalized to U48 and U49. *n* = 3; * *p* < 0.05 compared to control.

We provide evidence that ERα plays a key role in the regulation of miR-193a-3p. Since ERα has been previously shown to mediated regulation of miRNA at the transcriptional level [30,31], we examined the expression of the pri-miRNAs in HUVECs treated with E2. Our results show that E2 did not inhibit the expression of pri-miRNA after 6 and 24 h treatment (Figure 11), suggesting that E2 regulates miR-193a-3p procession rather than its transcription.

### 3.9. Methoxyestradiol, Endogenous E2 Metabolite Upregulates MiR-193a-3p Expression in HUVECs

**Figure 12:**
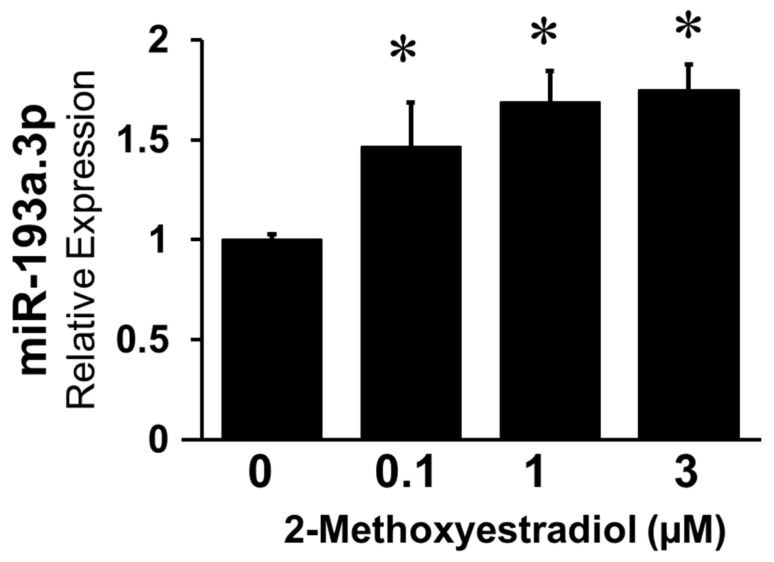
2ME induces miR-193a-3p expression in ECs. HUVECs were treated with 0.1–3 µM 2ME for 24 h, prior to RNA extraction and miR-193a-3p expression determination by qRT-PCR. The results are presented as mean ± SEM (*n* = 3). * *p* < 0.05 as compared to vehicle treated control.

In contrast to its effects on vascular smooth muscle cells (SMCs), the endogenous E2 metabolite 2-Methoxyestradil (2ME) has been shown to differentially regulate cellular functions in ECs, thus inhibiting proliferation and angiogenesis [32]. Hence, we investigated whether 2ME regulates miR-193a-3p in ECs. As shown in Figure 12, treatment of ECs with 2-ME upregulated the expression of miR-193a-3p, which may explain the differential effects of E2 and 2ME on EC growth and microvessel formation.

### 3.10. MiR-193a-3p Mimic Inhibits Basal and E2-Induced Angiogenesis In Vivo

Since E2 induced capillary formation and downregulated miR-193a-3p expression, together with the observation that miR-193a-3p mimic inhibited normal and E2-induced angiogenesis, we further assessed if miR-193a-3p downregulation mediates the angiogenic responses of E2 in vivo. Matrigel plugs containing E2, miR-193a-3p mimic (M193a), mir-mimic control (MC), or E2 plus/minus MC or M193a were implanted in ovariectomized female C57B1/6 mice, and the formation of capillaries was examined after 7 days.

**Figure 13:**
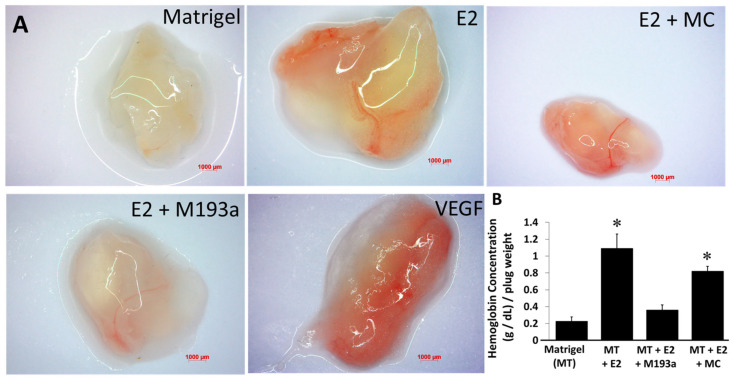
Inhibitory actions of miR-193a-3p (miRNA) on E2 (100 nmol/L)—induced angiogenesis/capillary formation. (**A**) Representative photomicrographs of Matrigel implants with vehicle (Matrigel negative control), E2, E2 + mimic control (MC), E2 + miR-193a-3p mimic (M193a), and VEGF (positive control) in ovariectomized mice. (**B**) Bar graph of hemoglobin content in Matrigel, a marker for angiogenesis. The levels of hemoglobin were significantly increased in plugs with VEGF (positive control), E2, and E2 + mimic control (MC), but not plugs treated with E2 + miR-193a-3p mimic (M193a). Data represent mean ± SEM from triplicate Matrigel plugs from three independent experiments; * *p* < 0.05 compared to MT alone.

As shown in Figure 13A, capillary formation was evident in implanted gels containing E2 and VEGF (positive control). Compared to E2 and VEGF, very little capillary formation was observed in plugs with Matrigel alone (negative control). Importantly, increased angiogenesis in response to E2 was blocked in plugs containing E2 + M193a, but not in plugs containing E2 + MC (Figure 13). Modulation of capillary formation by E2, as well as E2 combined with the M193a or MC group, was also associated with increased hemoglobin levels (Bar graph in Figure 13B), a marker for blood vessel formation. E2-induced capillary formation was accompanied with a significant increase in hemoglobin levels, and this effect was inhibited by M193a, but not by MC. Taken together, our results suggest that E2 and M193a mediate contrasting effects on capillary formation in vivo. Moreover, E2 potentially induces its angiogenic actions in ECs, in part, by downregulating miR-193a-3p expression in ECs.

### 3.11. E2 Modulates Circulating miR-193a Levels in Humans

**Figure 14:**
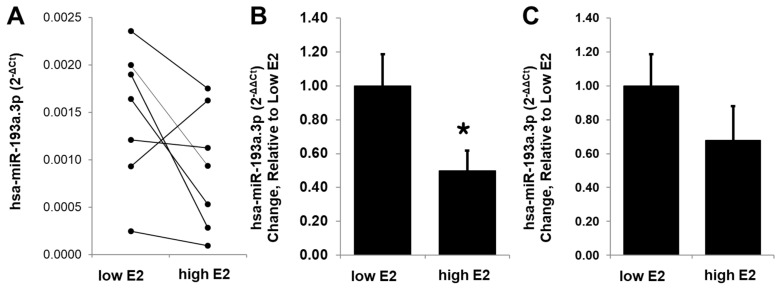
Circulating levels of miR-193a-3p decrease with the increase in E2 levels. Samples collected from 7 subjects during E2 production shutdown (low: <18–196 pmol/l E2 levels range) and during stimulation (high: 880–16314 pmol/l E2 levels range) were processed, and the levels of miR-193a-3p were quantified using RT-qPCR. (**A**) Mean changes in miR-193a-3p levels (*n* = 7). (**B**,**C**) Changes in miR-193a levels in the high E2 group relative to levels during the low E2 phase in the same patients following normalization of the individual low E2 values to 1. The results are presented as mean ± SEM (*n* = 7 in a group with the outlier and *n* = 6 in a group without the outlier). * *p* < 0.05 as compared to low E2 concentrations.

To extrapolate the potential relevance of our findings to humans, we assessed whether E2 is capable of modulating miR-193a-3p levels in humans. Using RT-PCR, we measured circulating miR-193a-3p levels in blood of women undergoing IVF treatment, where the levels of E2 were regulated to very low/negligible or high levels. As shown in Figure 14, miR-193a-3p levels were significantly higher during the low estrogen phase and decreased in all but one sample collected during the high E2 phase (Figure 14A). Since an increase in miR-193a was observed in just one subject, we assessed the relative change in miR-193a expression by excluding it as an outlier as well as including it. The trend for relative change in miR-193a in samples with high E2 was negative in samples with and without the outlier, with the percent change of 50 ± 12% (*n* = 6; *p* < 0.04) in a group without the outlier (Figure 14B) and 68 ± 0.20 in a group with the outlier (*n* = 7; *p* > 0.05; Figure 14C). Our findings suggest that the modulatory actions of E2 on miR-193a-3p may play a critical role in mediating the vascular protective actions of E2 and may represent a useful small molecule for potential therapeutic application.

## 4. Discussion

The protective effects of estrogens in the cardiovascular system are well established and include the promotion of endothelial repair and regeneration; however, the mechanisms involved remain unclear. The role of miRNAs in the vasculature has been extensively studied [18,19,20]. Recently, estrogens have been associated with alterations in miRNA expression [33,34], and several studies have shown that E2-regulated miRNAs mediate the actions of E2 in different tissues, including the vascular system [23]. Hence, the aim of this study was to assess the role of miRNAs, in particularly miR-193a-3p, in endothelial cell function and in mediating the protective effects of E2.

First, we examined the expression of four miRNAs (miR-193a-3p, miR-146a, miR-405, and miR-494). after E2 treatment in HUVECs. These miRNAs were chosen based on the literature research and our previous results from a screening of E2-regulated miRNA in endothelial progenitor cells (unpublished data).

Numerous studies have reported that miR-193a suppresses tumor development and inhibits tumor cell proliferation, migration, and invasion capabilities in different types of cancers [35]. Additionally, miR-193a seems able to limit proliferation and cell cycle progression in physiological contexts, in particular in endothelial colony-forming cells [36,37]. Moreover, previous studies from our lab demonstrated that miR-193a inhibits vascular EC function and that growth of vascular ECs in response to E2 was significantly downregulated in miR-193a-transfected cells, suggesting a role of miR-193a in E2-induced proliferation of vascular ECs [26].

Although miR-146a has been reported to exert controversial effects on both the inhibition and promotion of angiogenesis [38], there is extensive evidence that decreased miR-146a in HUVECs promoted angiogenesis [39]. Moreover, there is evidence for estradiol regulation of miR-146a [23], suggesting that regulation may play a role in E2-induced vasculogenesis.

For miR-409, there is evidence that it promotes retinal neovascularization in diabetic retinopathy [40]. Moreover, miR-409 is increased in patients with acute coronary syndrome and in a mouse model of acute myocardial ischemia. Moreover, overexpression decreases EC proliferation and migration, whereas inhibition has opposite effects, suggesting an important role of this miRNA in the angiogenic EC response to myocardial ischemia [40]. However, its role in E2-mediate vascular protection is still unknown.

Regarding miR-494, evidence indicates its decisive role in SMC proliferation and vascular remodeling [41] as well as inhibiting apoptosis [42]. Moreover, miR-494 was found to inhibit angiogenesis by targeting VEGF in HUVECs [43], while other studies reported that inhibiting miR-494 increases neovascularization and blood flow recovery after ischemia [44]. Interestingly, treatment with E2 significantly inhibited miR-494 expression in pericytes [45], and the upregulation of miR-494 has been shown to contribute to estrogen-mediated cardiovascular protection against oxidative stress [46], suggesting that this miRNA might play a role in promoting E2-induced EC regeneration.

Based on the above findings, all four miRNAs are interesting targets, since it is conceivable that the stimulatory effects of E2 on EC growth and motility are potentially mediated via their regulation. However, validation of miRNA altered expression after E2 treatment in HUVECs confirmed only downregulation of miR-193a by E2, while the expression levels of miR-146a, miR-409, and miR-494 were not altered. Considering that miRNA expression and function can be cell type-specific, it is feasible that miR-146a, miR-409, and miR-494 are differentially regulated by E2 in EPCs and HUVECs. Nevertheless, we confirmed previous results showing miR-193a downregulation by E2 in HUVECs [26].

The second aim of the study was to assess the role of miR-193a in EC function. Therefore, we transfected the cells with miR-193a mimics and antimir to upregulate and inhibit the miRNA expression, respectively. Interestingly, we found that miR-193a mimic inhibited cell growth, migration, and microvessel formation, while blocking the miRNA with the antimir significantly induced proliferation, wound closure, and angiogenesis, showing that miR-193a negatively regulates vascular function of endothelial cells and their ability to grow and migrate. Since E2 is known for its pro-growth and pro-angiogenic effects and it downregulates the expression of the EC function inhibiting miR-193a, we hypothesized that the protective effects of E2 may be mediated by downregulation of miR-193a. Indeed, the protective effects of E2 in HUVECs were blocked after transfection with miR-193a mimic, which significantly inhibited E2-induced microvessel formation and cell migration. These findings suggest that downregulation of miR-193a by E2 participates in the E2-mediated stimulatory effects on HUVECs. These results confirm previous findings from our lab showing that miR-193a inhibits serum-induced growth of vascular ECs and miR-193a-conditioned media from MCF-7 cells inhibit tube formation and migration and that growth stimulatory effects of E2 in both MCF-7 and vascular ECs were abrogated and reversed in cells transfected with miR-193a [26]. Moreover, other studies provide evidence for the growth-suppressing effects of miR-193a on vascular cells. In fact, Khoo et al. showed that miR-193a was highly expressed in the less proliferative peripheral blood endothelial colony-forming cells (ECFCs), where its inhibition using antimir molecules improved the in vitro proliferation, migration, and vascular tubule formation of these cells. miR-193a was expressed at low levels in the proliferative cord blood ECFS, where its overexpression limited cell cycle progression, migration, and angiogenesis [36].

Proliferation, migration, and vasculogenesis are all processes, which are influenced by cell viability, and their inhibition may result from increased apoptosis. miR-193a pro-apoptotic functions are reported in several tumors, including melanoma, hepatocellular carcinoma, acute myeloid leukemia, breast, and prostate cancer [47]. Overexpression of miR-193a induced activation of caspase 3/7 and resulted in apoptotic cell death of ovarian cancer cells [48]. However, in this study, we could not detect significant changes in cell viability after modulation of miR-193a, suggesting that the effects of miR-193a on EC function are not due to the influence of miR-193a on cell death. However, we used indirect methods to study the effect of the miRNAs on cell survival and did not specifically measure apoptosis. Therefore, the analysis of apoptosis using a specific marker, such as Annexin V or TUNEL staining, might give a better answer to how miR-193a influences EC function. Finally, the activity of miRNAs might strongly depend on the cell type and cellular environment; thus, it is feasible that, under our specific condition, miR-193a does not affect cell survival. Taken together, these findings suggest that miR-193a plays an important role in vascular remodeling and that the beneficial effects of E2 on the endothelium and consequently on the vascular system are in part mediated via downregulation of this miRNA and abrogation of its potentially disruptive impact in ECs.

To decipher whether miR-193a is indeed involved in E2-mediated proangiogenic actions in ECs, we next investigated its impact on key pathways via which E2 stimulates EC proliferation, migration, and vasculogenesis, such as PI3K/Akt signaling, VEGF, and the ALK1/SMAD1/5/8/ID1 pathway [26,28].

First, we looked at the modulation of Akt phosphorylation, a key signal transduction mechanism that feeds the downstream pro-angiogenic cascades involving VEGF. The importance of PI3K/Akt signaling in estrogen-mediated angiogenesis is well established [28,49]. Indeed, our findings that E2 induces Akt phosphorylation and tube formation in HUVECs and that these effects are blocked by the PI3K inhibitor LY294002 further reconfirm this contention. We found that miR-193a overexpression inhibits the phosphorylation of Akt. Moreover, treatment with the PI3K inhibitor LY294002 abrogated tube formation seen after miR-193a downregulation by the antimir, suggesting that miR-193a inhibits vasculogenesis by targeting the PI3K/Akt pathway.

The association of miR-193a and the PI3K/Akt pathway has been shown in a few studies. In acute myeloid leukemia, miR-193a indirectly inhibits phosphatase and tensin homolog (PTEN), thus activating Akt and contributing to leukemogenesis [50]. In addition, in macrophages, miR-193a overexpression led to increased Akt phosphorylation [51]. However, this is in contrast to the inhibitory actions we found in HUVECs. Other evidence of miR-193a association with PI3K/Akt has been shown in non-small-cell lung cancer, where it suppresses metastasis by targeting the receptor tyrosine-protein kinase ERBB4, a member of the epidermal growth factor (EGF) receptor subfamily, and inhibiting the downstream PI3K/Akt cascade [52]. Further, previous results from our lab additionally show that miR-193a inhibited extracellular signal-regulated kinase 1/2 (ERK1/2) and Akt phosphorylation in lymphatic ECs [53]. These findings suggest that miR-193a actions on PI3K/Akt signaling depend on the cell type. Moreover, we speculate that miR-193a targets molecules upstream of PI3K, such as ERBB4 or other receptor tyrosine-protein kinases, resulting in the inhibition of PI3K activity. Finally, there is evidence that miR-193a targets c-Kit in myeloid leukemia [54]. Thus, it is feasible that miR-193a might inhibit PI3K activity indirectly via suppressing stem cell factor (SCF)/c-Kit or VEGF signaling.

Downstream from activated Akt, nitric oxide (NO) and VEGF play major roles in mediating E2-induced pro-angiogenic actions in ECs [55,56,57]. Treatment with E2 has been shown to increase VEGF expression in vascular ECs [58]. Moreover, increased VEGF expression has been associated with E2-mediated endothelial recovery [10]. Indeed, both VEGF and NO are important mediators of estrogenic actions in ECs, and there is evidence that estrogen induced angiogenesis is dependent on VEGF and NO [59]. Here, we confirmed that treatment with E2 increases VEGF-A expression. In addition, treatment with a VEGF-specific neutralizing antibody (VEGFab) abrogated E2-induced vasculogenesis in HUVECs, confirming that VEGF indeed plays an important role in mediating the stimulatory actions of E2 in ECs.

In the current study, we provide the first evidence that miR-193a inhibits VEGF-A expression. Furthermore, our observations that vasculogenesis induced by the antimirs-neutralizing miR-193a is abrogated by VEGFab, suggest that VEGF plays a role in the regulation of tube formation by miR-193a in HUVECs. Little is known about the role of miR-193a in association with VEGF. Hua et al. found miR-193a-binding sites in the VEGF 3′-UTR, thus predicting that miR-193 could putatively target VEGF, based on bioinformatics algorithms. However, miR-193a was not differentially expressed in hypoxic compared to control nasopharyngeal carcinoma cells, and therefore, the association between miR-193a and VEGF was not further investigated [60]. Here, we demonstrate that miR-193a inhibits VEGF; however, whether this occurs via direct binding to its mRNA or via downregulation of the PI3K/Akt signaling pathway remains unclear and needs to be further investigated. The fact that PI3K/Akt induces NO synthase (eNOS), which in turn upregulates VEGF via NO, supports that the inhibitory actions of miR-193a on PI3K/Akt may contribute to their inhibitory effects on VEGF and thus tube formation. Indeed, inhibition of PI3K/Akt by LY blocks NO release, VEGF expression, and angiogenesis [61], and this may contribute to its abrogating actions on antimir-induced capillary formation.

Estrogens regulate TGF-β signaling [62], another prominent pathway in the regulation of EC growth, migration, and angiogenesis. Indeed, in our study, E2 increased the expression of ALK1 and ID1, a downstream target of SMAD1/5/8. Moreover, the ALK1-specific antagonizing antibody ALK1fc abrogated E2-induced angiogenesis, confirming previous findings from our laboratory, demonstrating that E2 mediates vasculogenesis and endothelial growth via the stimulation of ALK1/ID1 signaling [63,64].

Few reports link miR-193a and TGF-β; miR-193a/b overexpression significantly repressed TGF-β1 in hepatic stellar cells [65], and there is evidence that miR-193a stimulates pancreatic cancer cell repopulation and metastasis through modulating TGF-β2 [66]. However, the role of miR-193a in association with TGF-β in endothelial cells has not yet been reported. Here, we provide the first evidence that miR-193a inhibits the expression of both ALK1 and ID1, the downstream target of SMAD1/5/8. Moreover, miR-193a antimir-induced vasculogenesis was blocked in the presence of ALK1fc, providing evidence that ALK1/ID1 signaling is involved in the inhibitory actions of miR-193a. These findings suggest that miR-193 reduces angiogenesis via downregulation of the pro-angiogenic ALK1/ID1 signaling. Although there is no direct evidence for a role of miR-193a in this pathway, our findings are supported by the fact that, based on bioinformatics algorithms such as TargetScan [67] and miRIAD [68], ALK1 is a putative target of miR-193a. Therefore, it is feasible that E2 mediates angiogenesis by downregulating miR-193a.

**Figure 15:**
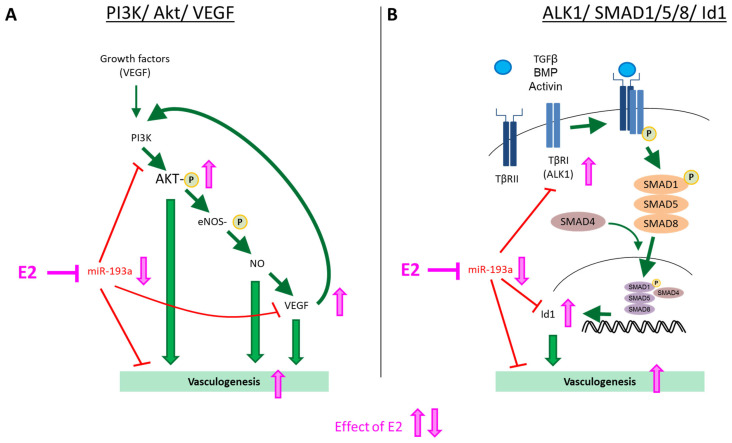
Schematic representation of the intracellular mechanisms by which miR-193a may mediate E2-induces vasculogenesis. (**A**) E2 promotes vasculogenesis by downregulating miR-193a expression, thus blocking miR-193a inhibitory actions on AKT-phosphorylation and VEGF expression. (**B**) miR-193a also inhibits the TFGβ signaling pathway. By reducing miR-193a expression, E2 reverses the suppressing effects of miR-193a on ALK1 and ID1 expression and induces microvessel formation.

Taken together, our findings suggest that miR-193a acts as an endogenous inhibitor for angiogenesis in ECs and mediates its actions by inhibiting PI3K/Akt, VEGF, and ALK1/ID1 signaling, both key pathways in E2-mediated pro-angiogenic function. Importantly, since E2 downregulates miR-193a, we suggest that E2 induces its pro-angiogenic actions by downregulating miR-193a expression and reversing its inhibitory actions on PI3K/Akt, VEGF, and ALK1/ID1 signaling (Figure 15).

The role of ERs in mediating the beneficial effects of E2 in vascular cells is well established. E2 has been shown to mediate its biological actions via nuclear and membrane ER α, β, and GPER/GPR30 [29]. Here, we provide the first evidence that ERα plays a key role in the regulation of miR-193a. Indeed, downregulation is mimicked by the ERα agonist PPT, but not by ERβ or GPER agonists. Moreover, the broad-spectrum ER antagonist ICI and the ERα specific antagonist MPP blocked E2- and PPT-dependent miRNA inhibition, suggesting that ERα is required for E2-mediated miRNA downregulation.

Numerous studies report that ERα mediates the cardiovascular protective effects of estrogen. In ERβ knock-out mice, estrogen was still protective against vascular injury [69], whereas in ERα knock-out mice estrogen treatment showed no protective effect [70], supporting that ERα is necessary. Although there is ample evidence for a role of ERα in mediating the vascular protective actions of E2, whether ER is responsible for the beneficial effect of E2 in the vascular system is still intensely debated. In support of our findings that ERα is required for downregulation of miR-193a, E2 and the ERα-selective agonist PPT decreased miR-206 expression in MCF-7 cells [71]. Moreover, Di Leva et al. reported that E2-ERα mediate the repression of miR-221/222 in MCF-7 cells [31], and Zhao et al. demonstrated that ERα mediates miR-203 upregulation by E2 in vascular SMCs and that miR-203 participates in the anti-mitogenic actions of E2 in these cells [30]. Interestingly, ERα has been shown to mediate the rapid E2 effects, leading to increased Akt phosphorylation in vascular ECs [72], and is required for the activation of eNOS and E2-dependent vascular relaxation in ECs [73], suggesting that E2 may induce its pro-angiogenic actions by downregulating miR-193a expression and reversing its inhibitory actions on PI3K/Akt and VEGF via ERα.

In summary, here, we provide evidence that E2-ERα is involved in miR-193a regulation; however, the molecular mechanisms by which E2-ERα inhibits miR-193a expression remain unknown. A relationship between ERα and miRNAs has been demonstrated and discussed, but direct proof for ERα elements within the promoter of miR-193a has not been clearly demonstrated [30]. Some findings also suggest that some members of the miR-193 family can target ERα [74]. It is also postulated that estrogen’s interaction with the miR-193a promoter is indirect, primarily through the regulation of transcription factors and co-regulatory networks that in turn influence mi-R193a expression. In this context, activation of ER signaling may increase E2F6, a transcriptional repressor which may induce epigenetic silencing of the miR-193a promoter, as has been shown for the upregulation of c-KIT [75]. Zhao and colleagues have shown that E2 upregulates the expression of 26 miRNAs and downregulates the expression of six miRNAs in mouse aorta. Importantly, transcription factors Zeb-1 and AP-1 play critical roles in mediating E2-induced upregulation of miR-203 via ERα [30]. Taken together, more in-depth studies are required to assess and confirm whether ERα elements are present within the miR-193a promoter.

ERα has been previously shown to mediate regulation of miRNA at the transcriptional level [30,31]. However, in contrast to our expectation, we found that E2 did not alter pri-miRNA expression, suggesting that E2 regulates miR-193a procession rather than its transcription. In support of our hypothesis, the relationship between estrogen action and miRNA biosynthesis has been extensively described in breast cancer samples, where differences in key miRNA-processing genes have been observed between ER+ and ER− breast cancer cells [76]. Moreover, mRNA microarray data of human EC treated with E2 reveled the downregulation of key miRNA biosynthesis pathway genes [77], and a more recent study from the same lab showed upregulation of DiGeorge Critical Region 8 (DGCR8) and downregulation of DICER1 and Argonaute RISC Catalytic Component 2 (AGO-2) proteins upon E2 treatment, suggesting that estrogen regulates endothelial miRNA production machinery [23]. Furthermore, there is evidence that E2-ERα directly inhibits pri-miRNA processing by Drosha, through interaction with p68 and p72, which are part of the Drosha microprocessor complex and established ERα coregulators [78]. Indeed, Drosha and p68/DDX5 could be co-purified with ERα in MCF-7 cells [79]. Additionally, Exportin-5, which controls the nuclear export of precursors, is induced by E2 [80], and processing of pre-miRNA to mature miRNA might also be coupled with ERα. Indeed, Dicer acts as a nuclear receptor coactivator in MCF-7 cells [81], and its expression was shown to be induced by E2 and is higher in Erα-positive breast cancers compared to negative ones [76,82]. Finally, there is contrasting evidence that the expression of AGO2, the catalytic component of the RNA-induced silencing complex (RISC), is regulated by E2 both negatively—through activation of EGF receptor/mitogen-activated protein kinase (MAPK) signaling [83]—and positively [76]. As E2 specifically inhibited miR-193a and did not affect the expression of the other investigated miRNAs, it is also feasible that E2 regulates miR-193a stability or decay. Indeed, a study from Kim et al. showed that E2 treatment can post-transcriptionally regulate the stability of mature miRNAs in hypothalamic-derived neuronal cell lines [84], thereby revealing a novel role of E2 in modulating mature miRNA behavior, independent of its effects on regulating the primary and/or precursor form of miRNAs.

Taken together, here, we provide the first evidence that E2 does not inhibit miR-193a transcription in HUVECs. Moreover, based on the recent findings that E2-ERα plays a role in miRNA production machinery and can regulate stability of mature miRNAs, we postulate that E2 downregulates miR-193a either by inhibiting their procession from primary transcript to mature functional miRNAs or by regulating miR-193a decay. However, the molecular mechanisms need to be further elucidated.

E2 metabolites are known to play a role in mediating its effects in vascular cells. In fact, 2ME mediates the protective action of E2 in vascular SMCs, while exerting differential effect in ECs, by inhibiting EC proliferation, migration, and angiogenesis [32] at higher or pharmacological concentrations. Although previous results from our lab show that E2 and 2ME differentially regulated miR-193a expression in EPCs (unpublished data), miR-193a has not yet been associated with 2ME. Our finding that growth inhibitory concentrations of 2ME upregulate miR-193a levels in ECs suggests that its anti-proliferative actions may, in part, be mediated via its stimulatory actions on miR-193a levels. Taken together, we provide the first evidence that 2ME induces miR-193a expression in HUVECs and speculate that miR-193a might modulate the differential antiangiogenic effects of the E2-metabolite. However, future in-depth research is necessary to test/investigate this hypothesis.

Our findings that miR-193a mediates the protective effects of estrogens in the cardiovascular system and that E2 promotes endothelial repair and regeneration by downregulation of miR-193a are further strengthened by our in vivo studies. We confirmed that E2-induced angiogenesis is inhibited by miR-193a in ovariectomized female C57B1/6 mice animal models. Indeed, increased capillary formation in response to E2 was abrogated in Matrigel plugs containing miR-193a mimic. These findings providing evidence that E2 induces angiogenic action in ECs by downregulating miR-193a expression. Nevertheless, the role of miR-193a in other vascular cells remains in its infancy. Although the Matrigel plug assay provides strong evidence that E2 induces its angiogenic actions by blocking miR-193a expression, the participation of other mechanism (s) or miRNAs cannot be ruled out and requires further in-depth investigation.

miR-193a might be the key miRNA that mediates the protective effects of E2 on vascular cells. Our preliminary experiments conducted in a limited number of subjects undergoing IVF treatment show that high levels of E2 correlate with decreased miR-193a expression in plasma samples. Although consistent with our observation on the effects of E2 on miR-193a levels in cultured HUVECs, the in vivo results need confirmation in a larger number of subjects. This contention is particularly important as the basal levels and E2-mediated changes in miR-193a varied considerably in subjects. However, we did observe an outlier, with a contrasting trend in miR-193a levels in one subject. It is feasible that the overall change in circulating levels may depend on EC health, presence of ERα, E2 metabolism, and presence of its metabolites, as well as other factors such as growth factors and pathological conditions. Hence, the possibility that there are responders and non-responders is high, as also observed for E2-induced NO responses [85]. All in all, our finding from cultured ECs and Matrigel plug assays in mice provides strong evidence that E2 regulates miR-193a in humans and that this regulation might play a role in angiogenesis, opening new options for human replacement therapies. However, miR-193a actions beyond the vascular system need to be further investigated, as miR-193a may be an important mediator of estrogen function also in other tissues and organs.

Although our findings provide evidence that downregulation of miR-193a by E2 induces EC growth and may protect the blood vessel, these actions may not be limited to the cardiovascular system. Angiogenesis associated with E2 is known to play a critical in follicle development, embryo implantation, and cancers/tumors (breast, uterine). The fact that E2 modulates the expression of miR-193a expression suggests that its deleterious or beneficial effects may be tissue-specific and should be interpreted accordingly.

In summary, we confirmed that E2 downregulates miR-193a expression in HVUECs through ERα and by either regulating miRNA biosynthesis or stability. Moreover, we confirmed the inhibitory action of miR-193a on endothelial cell proliferation, migration, and tube formation and that E2-mediated wound closure and angiogenesis are blocked by miR-193a overexpression, suggesting that miR-193a mediates the protective effects of E2. Further, we provide evidence that miR-193a inhibits angiogenesis in ECs by blocking PI3K/Akt, VEGF, and ALK1/ID1 signaling, both key pathways in E2-mediated pro-angiogenic function.

## 5. Conclusions

Our findings suggest that miR-193a may be interesting therapeutic targets in cardiovascular disease, to protect post-menopausal women against occlusive vascular disorders. Since miR-193a is expressed in both genders, they might also be useful to treat CVD in men. Moreover, miR-193a mimics could be also good target molecules as mediators of proangiogenic E2 effects in tissues with disrupted angiogenesis, while antimir molecules could be used to block unwanted vasculogenesis, for example for cancer treatment. However, it is yet unknown whether modulation of these miRNAs bypasses the cancerogenic side effects of hormones currently used for replacement therapy. Numerous studies revealed that it suppresses tumor development and migration in different types of cancers, including breast and ovarian cancers [35,37], suggesting that its role is cell- and tissue-type-dependent, and that further research is required to fully understand the roles of both miRNAs in the human body.

Compared to the classic molecular therapeutics, miRNA therapeutics is a promising field for the treatment of different disease. The function of miRNAs can be restored or inhibited through the delivery of synthetic miRNA mimics or antimirs, respectively, thus either blocking tumor proliferation and migration or restoring endothelial cell function in cardiovascular disease. However, several obstacles including miRNA stability, specific cell and tissue delivery, off-target effects, and the immunogenicity of both the nucleic acids and delivery vehicles still have to be resolved.

The successful use of miRNA therapeutics has been demonstrated against several cardiovascular and metabolic disorders and tumors. Indeed, a small number of miRNA inhibition and replacement strategies to restore original miRNA expression levels—including miR-193a mimic therapeutic for patients with advanced solid cancer [86]—have already entered preclinical and clinical trials. These approaches may be soon available for clinical use, suggesting that the modulation of miRNAs may be a promising technology for future therapeutic developments [87,88,89,90,91].

Limitations: Although our findings provide evidence that E2 mediates EC growth/angiogenesis by downregulating the expression of anti-angiogenic miR-193a-3p, involvement of other mechanism (s) and miRNAs in mediating the vascular protective actions of E2 cannot be ruled out. The observation that miR-193a levels are decreased in the majority of the subjects with high E2 levels is promising and reaffirms our in vitro observations. However, a low number of subjects, variation in basal miR-193 levels, and inconsistency in the trend and magnitude of effects remain limiting factors to reach a true conclusion. Hence, our findings in humans should solely serve as an important first step/lead in planning, assessing and confirming the impact of E2 on miR-193a levels in a larger number of subjects. Finally, our in vitro findings may not fully translate in an in vivo environment with potential complex factors in humans. For instance, inter-individual variation in multiple endogenous factors that regulate miRNA biogenesis, pathophysiology/health status, ER expression, and E2 metabolism may influence E2-mediated effects on miR-193a and angiogenesis.

## Data Availability

All data supporting the findings of this study are available within the article or from the corresponding author upon reasonable request.
